# Towards Understanding Sustained Neural Activity Across Syntactic Dependencies

**DOI:** 10.1162/nol_a_00050

**Published:** 2022-02-10

**Authors:** Aura A. L. Cruz Heredia, Bethany Dickerson, Ellen Lau

**Affiliations:** Department of Linguistics, University of Maryland, College Park, MD, USA; Department of Psychology, University of Pennsylvania, Philadelphia, PA, USA; Department of Linguistics, University of Massachusetts, Amherst, MA, USA

**Keywords:** sustained neural activity, working memory, language processing, syntactic dependencies, syntactic predictions, ERP, SAN

## Abstract

Sustained anterior negativities have been the focus of much neurolinguistics research concerned with the language-memory interface, but what neural computations do they actually reflect? During the comprehension of sentences with long-distance dependencies between elements (such as object *wh*-questions), prior event-related potential work has demonstrated sustained anterior negativities (SANs) across the dependency region. SANs have been traditionally interpreted as an index of working memory resources responsible for storing the first element (e.g., *wh*-phrase) until the second element (e.g., verb) is encountered and the two can be integrated. However, it is also known that humans pursue top-down approaches in processing long-distance dependencies—predicting units and structures before actually encountering them. This study tests the hypothesis that SANs are a more general neural index of syntactic prediction. Across three experiments, we evaluated SANs in traditional *wh*-dependency contrasts, but also in sentences in which subordinating adverbials (e.g., *although*) trigger a prediction for a second clause, compared to temporal adverbials (e.g., *today*) that do not. We find no SAN associated with subordinating adverbials, contra the syntactic prediction hypothesis. More surprisingly, we observe SANs across matrix questions but not embedded questions. Since both involved identical long-distance dependencies, these results are also inconsistent with the traditional syntactic working memory account of the SAN. We suggest that a more general hypothesis that sustained neural activity supports working memory can be maintained, however, if the sustained anterior negativity reflects working memory encoding at the non-linguistic discourse representation level, rather than at the sentence level.

## INTRODUCTION

A key aspect of human language is that syntactically codependent units in a sentence can appear at an arbitrarily long distance from one another. Previous behavioral work suggests that the parser pursues top-down structure-building strategies when processing these sorts of dependencies (e.g., Crain & Fodor, 1985; Stowe, 1986). Crucially, such a top-down approach requires maintaining a memory representation of predictively generated structures until they can be “checked” against the upcoming bottom-up input. How these predictions are represented online, however, is not well understood.

Consider (1) below. The *wh*-phrase *who* acts as the direct object of the verb *pleased* even though the two appear several words apart and in a non-canonical word order (i.e., the object precedes the verb). In contrast, in sentences like (2), the object and verb appear at linearly adjacent positions and in canonical word order. Processing long-distance dependencies like that between the fronted *wh*-phrase and the verb in (1) relies on working memory resources that can relate the two during comprehension. Moreover, evidence that the presence of a fronted *wh*-phrase initiates an active search for an object position ahead of encountering the head verb has been extensively reported in the sentence processing literature (e.g., Frazier & D’Arcais, 1989; Omaki et al., 2015).

(1)  Who did the compliments about the food please?
(2)  Did the compliments about the food please the chef?

While the neurocognitive mechanisms underlying this predictive structure-building computation are relatively underspecified, event-related potential (ERP) work on these types of structures has previously shown a sustained neural response over anterior electrodes for long-distance dependency conditions, relative to control conditions with either shorter or no nonlocal dependencies (e.g., Fiebach et al., 2002; Hagiwara et al., 2007; King & Kutas, 1995; Phillips et al., 2005). In the first such reported study, King and Kutas (1995) reported a *sustained anterior negativity* (SAN) for object relative clauses (RCs) like (3) in which a noun phrase (NP) has been moved outside of and away from its canonical position as the object of the main clause verb, relative to subject RCs like (4). The authors attributed the SAN to the *working memory* costs associated with storing and maintaining the filler in memory throughout the dependency. This finding resembles the germinal report by Fuster and Alexander (1971) showing that subpopulations of neurons in primates’ prefrontal cortex fire continuously when a target stimulus needs to be stored and maintained online across a delay period (in the absence of the stimulus itself) in a working memory task. While such a functional interpretation of persistent neural activity is not without debate (see Constantinidis et al., 2018, and Lundqvist et al., 2018, for dual perspectives; see D’Esposito & Postle, 2015, for a review), sustained activity during working memory tasks has been reported across a number of studies using a variety of methodologies.

Although prior studies on sentence processing have provided evidence that *long-distance dependencies* are associated with sustained neural processing differences, exactly what kind of working memory computation is indexed by the SAN remains uncertain. In this study, we set out to address this question directly by testing the hypothesis that what is being encoded and maintained available via *sustained neural activity* is specifically the representation of predictively generated structures until they are confirmed by eventual bottom-up input. As detailed below, this hypothesis is in line with behavioral work showing that comprehenders actively construct these relations as soon as the long-distance dependency is identified, and before encountering information that confirms them (e.g., Omaki et al., 2015). Our approach was to evaluate sustained ERP responses to subordinate adverbial constructions (*After Charlotte quit* …), which require syntactic prediction but are substantially different from *wh*-dependencies in other relevant dimensions.

### Background

As mentioned above, one of the first and most influential reports that encoding long-distance dependencies may engender sustained neural activity comes from ERP work by King and Kutas (1995). These authors compared ERPs to RCs like (3) and (4) (The examples in (3) and (4) are from King & Kutas, 1995, p. 380). In (3), the object NP *the reporter* has been moved from its canonical position as the direct object of the verb—*attacked*—to the beginning of the main clause, creating a long-distance dependency between the object and verb. In (4), the reporter acts instead as the subject of the RC verb, creating a much shorter dependency between the NP and the verb. The authors showed a sustained negative ERP with an anterior distribution (i.e., SAN) for object RCs relative to subject RCs. The response began at the moved object-NP and lasted throughout the dependency. King and Kutas (1995) attributed this effect to the memory operations associated with storing and maintaining the filler in memory until its role as the object of the RC could be assigned upon encountering the RC verb.

(3)  The reporter_i_ who the senator harshly attacked ___i_ admitted the error.
(4)  The reporter_i_ who ___i_ harshly attacked the senator admitted the error.

A similar result from a study with more closely matched conditions was reported by Fiebach et al. (2002), who studied subject and object *wh*-dependencies in German—a verb-final, case-marked language in which the thematic role of moved elements can be assigned ahead of any specific verb-level information. This means that the subject/object gap distinction can be manipulated simply by changing the case marker on the leading *wh*-phrase. Fiebach et al.’s study was designed, in part, to evaluate (i) whether the SAN could be attributed to working memory processes irrespective of any specific syntactic/semantic operations (c.f., King & Kutas, 1995) and (ii) whether the SAN is sensitive to the length of the dependency. The main finding reported was that only the long object *wh*-dependencies (in which the *wh*-object and verb were separated by four prepositional phrases, as shown in (5)) elicited a SAN, relative to word- and dependency-matched subject *wh*-questions (6). (The examples in (5) and (6) are from Fiebach et al., 2001, p. 324, Table 1.) The authors attributed the SAN to working memory processes required for maintaining the dislocated object filler in memory (regardless of the early assignment of thematic roles) and argued that the maintenance process has a progressively increasing cost across the length of the dependency (as reflected by an amplitude increase in the SAN), as suggested in Gibson’s (1998) model of memory cost in sentence processing. However, Phillips et al. (2005) found evidence against this latter claim.

(5)  Thomas fragt sich, wen_i_ am Dienstag nachmittag nach dem Unfall der Doktor ___i_ verständigt hat.
    Thomas asks himself, who_(ACC)_ on Tuesday afternoon after the accident the_(NOM)_ doctor called has.
(6)  Thomas fragt sich, wer_i_ ___i_ am Dienstag nachmittag nach dem Unfall den Doktor verständigt hat.
    Thomas asks himself, who_(NOM)_ on Tuesday afternoon after the accident the_(ACC)_ doctor called has.

Phillips et al. (2005) conducted an ERP study of *wh*-dependencies aimed at clarifying whether the cost of holding incomplete dependencies in memory is length-sensitive. Gibson’s (1998) model of sentence processing proposed that the cost of maintaining an open syntactic dependency in memory actually increases across the course of the dependency. Phillips et al.’s logic for testing this idea was as follows: If the amplitude of the SAN reflects the storage cost of holding an incomplete dependency in working memory, and if this cost increases across the dependency, then later intervals of the dependency (i.e., later words) should show larger anterior negativities for the *wh*-condition relative to controls (Phillips et al., 2005, p. 415). Furthermore, this amplified negativity should be larger for longer *wh*-dependencies. To measure the individual contribution of single words to the amplitude of the SAN, the authors conducted a noncumulative analysis in which mean voltages were computed relative to a baseline at the beginning of each individual word. These differences were then compared between short (one-clause) and long (two-clause) *wh*-dependencies. Phillips et al. (2005) found that later words in the long *wh*-dependency made little contribution to the overall amplitude of the sustained effect and thus concluded that the SAN does not provide evidence of a length-sensitive cost in the processing of nonlocal syntactic dependencies. Importantly, while this result suggests that the SAN is not length-sensitive, the presence of a SAN for both short and long dependencies provides evidence that the effect is dependency-sensitive.

Evidence supporting a functional interpretation of the SAN as an index of working memory operations can be found in a number of other studies that vary across task, stimuli, and modality (e.g., Alunni-Menichini et al., 2014; Barkley et al., 2015; Lefebvre et al., 2013; Sprouse et al., 2021; Ueno & Kluender, 2003; Yano & Koizumi, 2018), although it is worth noting that at least one study of *wh*-dependencies failed to observe a SAN (Kaan et al., 2000). But what exactly is the working memory operation driving the positive effects? One idea is that the neural cost indexed by the SAN is the cost of maintaining the filler in working memory, keeping it accessible for the moment when the long-distance dependency relation can be formed.

As noted in the Introduction, neuroscientists have long explored the idea that keeping material accessible in working memory requires neurons responsible for encoding the representation to fire continuously (see D’Esposito & Postle, 2015, for a review). In the case of sentence processing, this has typically been thought of as maintaining the filler or its properties in memory throughout a dependency. However, a slightly different idea is that it is the maintenance of a syntactic prediction itself that requires continuous firing, until that prediction can be confirmed by the bottom-up input. As noted earlier, a large body of psycholinguistic work has demonstrated that long-distance dependencies are processed predictively: Certain sentential relations can be constructed ahead of the bottom-up input (Gibson, 1998). Either of these accounts (filler maintenance or syntactic prediction) then predicts that, if maintenance of information in working memory is implemented through sustained neural activity, we should observe an increase in neural activity beginning at the start of the dependency, and extending throughout it.

Some support for the syntactic prediction account comes from Fiebach et al. (2002). In their study on German *wh*-dependencies, Fiebach et al. found that case-marked and length-matched embedded object *wh*-questions ((5) above) elicited a SAN relative to subject *wh*-questions ((6) above). This result is puzzling if maintenance of the filler is what drives the SAN response, since the distance between filler and gap is identical in the conditions. However, the amount of structure that needs to be predicted in the two conditions differs: In the *wh*-subject items only a verb needs to be predicted, while in the *wh*-object items an extra argument (namely, an object position) needs to be predicted ahead of the verb. The presence of a SAN for the German object/subject *wh*-contrast is therefore compatible with a view in which neural activity increases with the amount of information predicted ahead of the bottom-up input.

In the present study, we evaluated SANs for sentences with filler–gap dependencies as well as sentences without filler–gaps that vary in the amount of predicted structure. To foreshadow our results: Across three experiments we found renewed evidence for the presence of a SAN in some kinds of *wh*-dependencies, but we failed to systematically observe SANs across all contrasts where syntactic prediction was modulated. On the basis of our results, we will conclude that the SAN is not simply a general index of maintaining syntactic predictions, or filler material. We will highlight instead pragmatic/interpretative processes involved throughout the dependency as candidate drivers for the SAN, in line with results from Yano and Koizumi (2018, 2021).

### The Current Study

**Figure 1.**
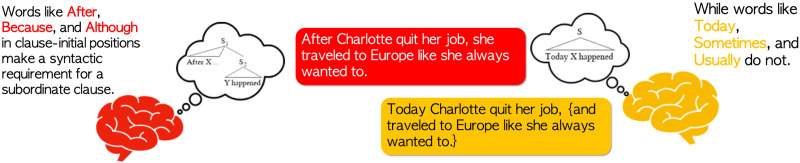
Cartoon depiction of what is meant by *syntactic prediction* in the subordinating adverb condition.

The goal of this study was to begin addressing what representations are encoded in working memory during the processing of nonlocal syntactic dependencies, as indexed by the SAN. Here, we wanted to assess whether the SAN is best characterized by a syntactic-prediction account, whereby an increase in the amount of structural prediction elicits sustained increases in neural activity. We did this by testing the response to sentences that vary the amount of syntactic prediction in the absence of a filler–gap dependency ((9, 10) below), alongside a more classic SAN filler–gap contrast ((7, 8) below). (The sentences in examples (7) and (8) are from Sprouse et al., 2021.) Specifically, we compared sentences with subordinating adverbials (like *Although*, *Because*, and *Whenever*), which trigger the prediction of a matrix clause following a subordinate clause, to sentences with non-subordinating adverbials (like *Today*, *Yesterday*, and *Sometimes*), which make no additional structural demands (see Figure 1 for a cartoon schematic). We predicted that, if the SAN is an index of actively maintaining nonlocal syntactic predictions, subordinating adverbs like (9) should show a SAN relative to sentences with non-subordinating adverbs like (10). We conducted three experiments to test this hypothesis.

(7)  What did the commentary from the spokesman interrupt?
(8)  Did the commentary from the spokesman interrupt the game?
(9)  After Charlotte quit her boring job, she traveled to Europe like she always wanted to.
(10)  Today Charlotte quit her boring job (and traveled to Europe like she always wanted to).

Experiment 1 evaluated the presence of a SAN for relatively short and simple *wh*-dependencies in order to replicate prior studies. Stimuli were matrix object *wh*-questions (3) and *yes*/*no* questions (4), which have no long-distance dependencies. Experiment 2 evaluated the SAN for embedded *wh*-questions and the critical adverb contrast within the same participants. We predicted a SAN for object *wh*-questions relative to controls and for sentences with subordinating adverbials relative to temporal controls if the SAN indexes syntactic predictions per se. Finally, Experiment 3 was conducted to replicate and extend the findings of Experiments 1 and 2. There, we evaluated the SAN for the matrix *wh*-dependencies and the critical adverb contrast within the same subjects. The predictions were the same as for Experiment 2.

## MATERIALS AND METHODS

### Experiment 1

The goal of Experiment 1 was to confirm that we could obtain the SAN for *wh*-dependencies in prior work. We used materials from a recent study by Sprouse and colleagues (2021) which observed a SAN in response to *wh*-dependencies in simple, one clause sentences.

#### Materials

Experiment 1 contained two conditions: a *wh*-dependency condition (7) and a polar (*yes*/*no*) question condition (8) that has no long-distance dependencies.

(7)  What did the commentary from the spokesman interrupt?
(8)  Did the commentary from the spokesman interrupt the game?

We created 60 item pairs based on a subset of items from Sprouse et al. (2021), each pair composed of a *wh*-dependency question and a corresponding *yes*/*no* question. Sentences in the *wh*-condition were always matrix object *wh*-questions, and the intervening subject phrase always contained five words in the form determiner-noun-modifier phrase. Half of the item sets contained a *who* question and half contained a *what* question. Sentence pairs were word-matched from the auxiliary (*did*/*Did*) to the verb (e.g., *please*); in *yes*/*no* questions the verb was always followed by an object NP such that the sentence as a whole was grammatical.

In this experiment, participants were asked to evaluate whether a follow-up probe sentence was a felicitous response to the target question or not (for each question in a pair we also created a follow-up sentence). Half of the follow-ups were felicitous, and half were not (see Procedure below for more details and examples).

Pairs of sentences were distributed across two lists following a Latin square design, such that each participant saw 30 trials per condition for a total 60 sentences, and no individual subject saw both versions of the same sentence. The two lists were pre-randomized.

#### Procedure

Question stimuli were presented visually, one word at a time, in white Arial font (24-point case), against a black background. Each trial began with a 900 ms fixation cross, followed by a 200 ms blank screen. Each word was displayed for 300 ms with an interstimulus interval of 200 ms (blank screen), for a total stimulus onset asynchrony of 500 ms. The final word of each question contained a question mark (e.g., *please?*) and was displayed on the screen for 400 ms, followed by a 200-ms blank screen before displaying the follow-up sentence. Items varied in overall length between 8 and 10 words, or 4.4–5.4 sec.

After each critical question stimuli, a follow-up probe was displayed on the screen in full until a response was provided. Participants were told that they should evaluate whether the follow-up made sense as a response to the question that preceded it. For example, after seeing (7) above, subjects might see the follow-up *The compliments pleased the chef*, which would be considered a good follow-up. Conversely, if they saw *The chef was very disappointed with her staff* as a follow-up, then this would be considered a bad follow-up, as it does not appropriately answer/address the question being posed. Responses were made using the F (“makes sense”) and J (“doesn’t make sense”) keys on a standard computer keyboard. The experiment itself was self-paced (subjects could decide when to begin the next trial).

A practice session consisting of 10 unique trials preceded the experiment to familiarize subjects with the experiment format and task. Participants were given feedback on each practice question, explaining which response they should have given, and why. Participants were encouraged to wait until the end of each trial to blink (i.e., not blink during flashing words), and were told that they could take breaks in between (but not during) trials.

As the current experiment was quite short in duration (∼25 min), it was combined in the same session with a separate ERP sentence experiment on an unrelated topic (argument structure). The current experiment was always conducted in the second part of the session. The two experiments were clearly delineated as separate, having different instructions, different practice sessions, and different stimuli format and task. (The other experiment had only one declarative sentence per trial, and compared the response to canonical and role-reversed sentences using a plausibility judgment task.)

#### Participants

A total of 21 participants (12 female, 19–31 years old, mean age 21.4 years) were recruited for the first experiment. Participants were compensated with university course credit or pay. All participants were native speakers of English (exposed before age 5), and all were classified as being right-handed by the Edinburgh Handedness Inventory (Oldfield, 1971).

Participants were included in our critical analysis if they had less than 30% of their trials rejected during pre-processing due to artifacts (i.e., at least 70% artifact-free trials) and had at least 60% accuracy on the comprehension task. A total of 7 participants were excluded based on these criteria, leaving 14 participants (6 female, 19–31 years old, mean age 22.3 years, all right-handed) for analysis. This number of participants is comparable to previous work on SANs (e.g., Phillips et al., 2005, *n* = 16).

#### EEG data acquisition

Twenty-nine tin electrodes (O1, O2, P7, P3, Pz, P4, P8, TP7, CP3, CPz, CP4, TP8, T7, C3, Cz, C4, T8, FT7, FC3, FCz, FC4, FT8, F7, F3, Fz, F4, F8, FP1, FP2) were held in place on the scalp by an elastic cap (Electro-Cap International, Inc., Eaton, OH). Bipolar electrodes were placed above and below the left eye and at the outer canthus of the right and left eyes to monitor vertical and horizontal eye movements. Additional electrodes were placed over the left and right mastoids. Scalp electrodes were referenced online to the left mastoid and re-referenced off-line to the average of left and right mastoids. The ground electrode was positioned on the scalp in front of Fz. Impedances were maintained at less than 5 kΩ for all scalp and ocular electrode sites and less than 3 kΩ for mastoid sites. The EEG signal was amplified by a NeuroScan SynAmps® Model 5083 (NeuroScan, Inc., Charlotte, NC) with a bandpass of 0.05–100 Hz and was continuously sampled at 500 Hz by an analog-to-digital converter.

#### EEG analysis

Epochs for the *wh*-contrast were time-locked to the onset of the determiner (*the*) immediately following the auxiliary (*did*) in both conditions and covered a span of −100:2,500 ms (i.e., extending through the second noun, up to but excluding the verb) and were baselined to the −100:0 ms pre-stimulus interval. We chose to time-lock and baseline to the onset of the determiner, following Sprouse et al. (2021), who note that time-locking and baselining to the preceding auxiliary could result in baseline artifacts due to the fact that this auxiliary was sentence-initial in one condition and not the other. Like them, we chose not to include the verb region in the analysis window since activity in this region may reflect termination of the dependency instead of or in addition to maintenance.

Pre-processing was done using the artifact detection routines provided by the EEGLAB (Delorme & Makeig, 2004) and ERPLAB (Lopez-Calderon & Luck, 2014) toolboxes. Eye-blink and eye-movement artifacts were identified using the ERPLAB step-function routine (Lopez-Calderon & Luck, 2014) with VEOG threshold of 40 Hz and an HEOG threshold of 25 Hz. Muscular and alpha wave artifacts were identified using the peak-to-peak routine with thresholds ranging between 100 and 110 Hz, based on visual inspection. When necessary, channels were spherically interpolated on a per-participant basis using the eeg_interp() function from the EEGLAB toolbox (Delorme & Makeig, 2004). We rejected approximately 5.7% (9 items) of all trials (4.4% WH; 3.3% YN) for the 14 participants included in the analysis. ERPs were formed off-line from clean epochs after pre-processing and artifact rejection, and a 40-Hz low-pass filter was applied. A table containing the specific pre-processing parameters used per participant can be found in the Supplementary Materials. (Supporting Information can be found at https://doi.org/10.1162/nol_a_00050).

To evaluate the presence of a SAN, we conducted a 2 × 2 (condition: WH/YN × anteriority: anterior/posterior) repeated-measures ANOVA on a subset of 20 electrodes, those 10 in the two forward-most rows of electrodes (FT7, FC3, FCz, FC4, FT8, F7, F3, Fz, F4, F8) and those 10 in the two backward-most rows of electrodes (P7, P3, Pz, P4, P8, TP7, CP3, CPz, CP4, TP8). We expected a SAN to manifest as an interaction between sentence condition and electrode site, such that anterior electrodes appeared more negative for the *wh*-condition relative to the control condition.

Statistical analyses were conducted on data from a time-window that began 750 ms after the onset of the word that signaled the dependency (i.e., *did* in both conditions). We chose this time-window based on the time course of the SAN response observed in a prior experiment by our collaborators (Sprouse et al., 2021). Statistical analyses were conducted using the ezANOVA package in R (R Core Team, 2013). In all experiments in the article we will report all main effects and interactions involving the factor condition, but as is standard we will not report simple effects of electrode position as we have no hypotheses about the distribution of scalp voltages independent of condition.

#### Experiment 1 results

**Figure 2.**
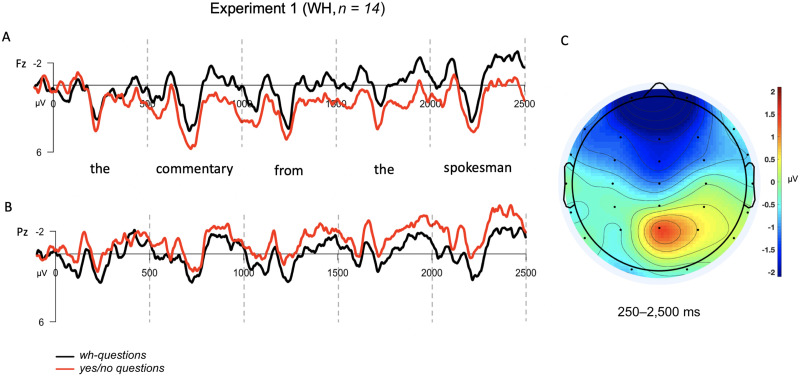
Grand averaged (GA) results for the matrix *wh*- contrast used in Experiment 1. The epoch is time-locked to the determiner (*the*) and spans 2,500 ms into the dependency (up to but excluding the verb). (A) GA waveform measured at frontal-central electrode, Fz. (B) GA waveform measured at posterior-central electrode, Pz. (C) Topographic scalp voltage map for region analyzed (250–2,500 ms), showing the voltage difference between *wh*- and *yes*/*no* question conditions (WH − YN). The scale is −2 μV (blue) to +2 μV (red).

The overall mean behavioral accuracy was 87.5% (WH: 87%, 10% *SD*; YN: 88%, 6% *SD*). As shown in the ERPs (Figure 2), we observed a negativity for the *wh*-question condition over the *yes*/*no* question in anterior electrodes that sustained across the epoch, mirrored by the opposite pattern in posterior electrodes. The ANOVA revealed a significant interaction between the two factors in the predicted direction (*F*(13) = 8.12, *p* = 0.01): Anterior sites were significantly more negative for the *wh*-condition (*M*_WH_ = 0.20 μV) than the *yes*/*no* condition (*M*_YN_ = 1.27 μV); the opposite was true at posterior sites (*M*_WH_ = −0.84 μV, *M*_YN_ = −1.04 μV).

#### Experiment 1 discussion

In Experiment 1, we observed reliable differences in the ERP response across the course of a *wh*-dependency relative to a control condition, as in prior work (e.g., Fiebach et al., 2002; Phillips et al., 2005). We saw a long-lasting increased anterior negativity in *wh*-questions relative to *yes*/*no* questions that began shortly after the *wh*-sentence was disambiguated to a (nonlocal) object question. These results straightforwardly replicate recent results from Sprouse et al. (2021) that use similar stimuli, and are in line with the earlier work reporting SANs for nonlocal *wh*-dependencies. This experiment confirms that our own recording and analysis parameters are appropriate for identifying SANs of the type classically reported in the sentence-processing literature.

### Experiment 2

The goal of Experiment 2 was to assess whether a SAN could be elicited by subordinate-matrix sentences that instantiate a syntactic prediction versus control sentences that do not.

#### Materials

(11)  After Charlotte quit her boring job, she traveled to Europe.
(12)  Today Charlotte quit her boring job and traveled to Europe.
(13)  John asked who the compliments about the food pleased.
(14)  John asked whether the compliments about the food pleased the chef.

Experiment 2 contained four conditions in a 2 (prediction) × 2 (sentence type) design: a subordinating adverb condition (11), a control non-subordinating (henceforth, *temporal adverb*) condition (12), a *wh*-dependency condition (13), and a control non-dependency (henceforth, *whether*) condition (14). These stimuli were chosen because they offer a simple case of syntactic prediction and are relatively easy to word-match across conditions. A *wh*-contrast (13, 14) similar to the one in Experiment 1 was included in order to localize the SAN response within the same participants. Thus, the experiment had two main contrasts: the critical adverb contrast and a *wh*-contrast. There were 30 trials per condition and 60 unrelated filler items for a total of 180 sentences read per participant. Stimuli were arranged following a Latin square design, and pairs were divided into two lists and pre-randomized as in Experiment 1. These two lists were then randomized again to create a total of four lists.

Sentences in the subordinate condition always started with a subordinating adverbial (e.g., *After*) and had two clauses. Structurally, the adverb cues the prediction of an obligatory second (matrix) clause following the subordinate clause (e.g., *After Charlotte quit her job). In contrast, control sentences—which were indicated by a non-subordinating temporal adverbial (e.g., *Today*)—did not instantiate a requirement for two clauses (e.g., *Today Charlotte quit her job*).

To better match the structures of the *wh*-contrast sentences to those used for the adverb contrast, we used embedded (indirect) *wh*-questions (and embedded controls), as shown in (13, 14). Importantly, the nonlocal *wh*-dependency in the embedded context is structurally the same as in the matrix context (Experiment 1), and as such both should elicit the same predictive and dependency-processing mechanisms.

Given the nature of the sentence stimuli used, subjects in Experiment 2 were asked to respond to a two-answer forced choice comprehension probe. Probes were presented after half of the trials and varied with respect to topic. For example, after seeing (11) or (12) above, subjects could be given a probe like *Who quit their job?* and given the options *Mindy* and *Charlotte*. Alternatively, they could be given a question such as *Where did Charlotte go?* with the options *Europe* and *Asia*.

#### Procedure

All presentation parameters were the same as in Experiment 1. Participants completed five practice trials using sentences unrelated to the experimental manipulation to familiarize them with the comprehension task. Three of these trials were followed by comprehension probes, and participants were given feedback on their performance.

#### Participants

A total of 30 participants (18 females, 18–24 years old, mean age 20.1 years) were recruited for Experiment 2. Participants were compensated with university course credit or pay. All participants were native speakers of English (exposed before age 5), and all but one participant (who was ambidextrous) were classified as being right-handed by the Edinburgh Handedness Inventory (Oldfield, 1971). Inclusion criteria were the same as before (at least 70% of artifact-free trials and 60% overall behavioral accuracy). 7 participants were excluded based on these parameters and 1 due to experimenter error, leaving 22 participants (12 females, 18–24 years old, mean age 19.9 years, all but one right-handed, one ambidextrous) for analysis.

#### EEG data acquisition

EEG data acquisition procedures were identical to Experiment 1.

#### EEG analysis

As in Experiment 1, epochs for the *wh*-contrast were time-locked to the onset of the *wh*-word in each condition and were extracted from −100:2,500 ms, which corresponded to the full dependency up to, but excluding, the verb. For the adverb contrast, epochs were time-locked to the onset of the adverb in both conditions and were extracted also from −100:2,500 ms, which corresponded to the entire first clause. Therefore, in both test cases, the epoch always spanned from the start of the dependency, until the word preceding the end of the dependency (the verb for *wh*-, the start of the second clause for adverbs).

Averaged ERPs were formed off-line from clean epochs after pre-processing and artifact rejection. Eye-blink and eye-movement artifacts were identified using the ERPLAB step-function routine with a VEOG threshold of 40 Hz and a HEOG threshold of 25 Hz. Muscular and alpha wave artifacts were identified using the peak-to-peak routine with thresholds ranging between 100 and 135 Hz, based on visual inspection. When necessary, channels were interpolated on a per-participant basis. We rejected approximately 18% (21 items) of all trials: 15.3% for the *wh*-contrast (15.8% WH, 14.8% YN) and 20.6% for the adverb-contrast (20.9% SUB, 20.3% TEMP). For both contrasts, a 100 ms pre-stimulus baseline and a 40 Hz low-pass filter were applied to the waveforms off-line. A list of the specific pre-processing parameters used per participant can be found in the Supplementary Materials.

To evaluate the presence of a sustained effect at anterior electrodes, we followed the same analysis plan outlined for Experiment 1. Again, the ANOVA was conducted on the time-window beginning 750 ms after the word triggering a dependency (*who*/*what*) or the corresponding position in the control conditions (*whether*) and spanned through the end of the epoch (i.e., 750–2,500 ms).

#### Experiment 2 results

Overall mean behavioral accuracy was 96% with 4% *SD* (WH: 99%, 1% *SD*; YN: 94%, 4% *SD*; SUB: 94%, 5% *SD*; TEMP: 93%, 6% *SD*).

**Figure 3.**
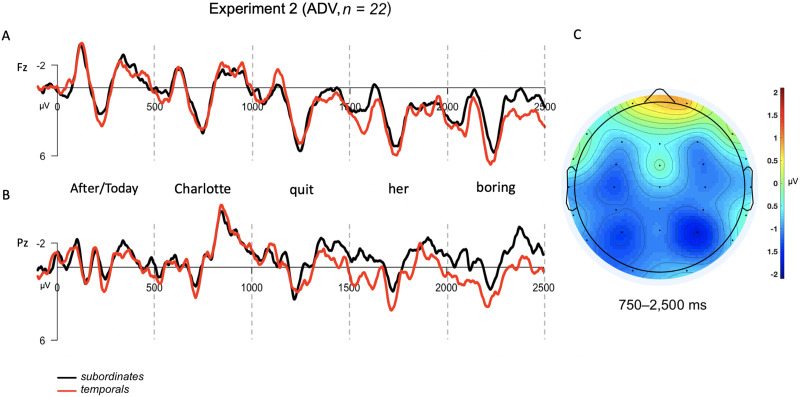
Grand averaged (GA) results for adverb contrast tested in Experiment 2. The epoch is time-locked to the adverb in each condition (*After*/*Today*) and spans 2,500 ms into the dependency (up to but excluding the second clause). (A) GA waveform measured at frontal-central electrode, Fz. (B) GA waveform measured at posterior-central electrode, Pz. (C) Topographic scalp voltage map for region analyzed (750–2, 500 ms), showing the voltage difference between subordinates and temporal adverb conditions (SUB − TEMP). The scale is −2 μV (blue) to +2 μV (red).

For the critical adverb contrast, a repeated-measures ANOVA with the parameters described above revealed no significant main effect of condition (*F*(21) = 1.63, *p* = 0.22) and no significant interaction between condition and anteriority (*F*(21) = 0.99, *p* = 0.33). Thus, as observable in Figure 3, we failed to detect a SAN in the subordinate condition relative to control (*M*_SUB,Ant_ = 1.25 μV; *M*_SUB,Post_ = −0.79 μV; *M*_TEMP,Ant_ =1.82 μV; *M*_TEMP,Post_ = 0.22 μV).

**Figure 4.**
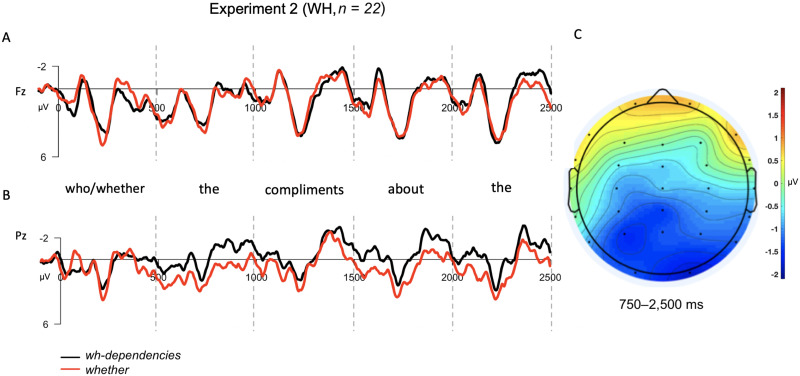
Grand averaged (GA) results for the embedded *wh*-contrast used in Experiment 2. The epoch is time-locked to the start of the dependency in the *wh*-condition and the corresponding word (*whether*) in the control condition, and spans throughout the dependency in the *wh*-condition and spanning 2,500 ms as in Experiment 1. (A) GA waveform measured at frontal-central electrode, Fz. (B) GA waveform measured at posterior-central electrode, Pz. (C) Topographic scalp voltage map for region analyzed (750–2,500 ms), showing the voltage difference between *wh*- and control condition (WH − WHETHER). The scale is −2 μV (blue) to +2 μV (red).

Notably, for the embedded *wh*-contrast, the results of this experiment patterned differently than those reported in Experiment 1. While there was still a significant interaction between the sentence type and electrode site factors (*F*(21) = 4.83, *p* = 0.04), the interaction was due to more negativity for the *wh*-condition at posterior electrodes (*M*_WH,Post_ = −0.91 μV, *M*_WHETHER,Post_ = 0.19 μV), while anterior electrodes showed nearly identical response values for both conditions (*M*_WH,Ant_ = 0.84 μV, *M*_WHETHER,Post_ = 0.83 μV). See Figure 4.

#### Experiment 2 discussion

In Experiment 2, we evaluated the hypothesis that SANs are a general index of syntactic prediction by investigating whether a SAN is elicited by subordinate sentences that required the prediction of a subsequent matrix clause, relative to control sentences that did not. We also included a separate control comparison designed to elicit the standard *wh*-dependency SAN, this time using embedded *wh*-s. We failed to find evidence for the hypothesis that SANs index syntactic predictions: ERPs to subordinate sentences showed no significant differences from ERPs to control sentences across the time-course of the initial clause in which the prediction must be maintained. According to the logic of our original design, this would suggest that SANs must index a different component of *wh*-dependency processing, for example, memory maintenance of the properties of the filler or interpretive computations specific to *wh*-dependencies.

However, the interpretation of these results is somewhat complicated by the surprising finding that the *wh*-contrast in Experiment 2 did not show evidence of a SAN either. Instead, ERPs showed a sustained increased negativity for *wh*-dependencies at posterior, rather than anterior, electrodes. Thus, while a sustained response was observed for *wh*-dependency sentences, a SAN was not. The lack of a SAN for embedded *wh*-sentences was surprising given that the syntactic dependency between filler and gap should not be altered by embedding, although it is worth noting that a previous ERP study by Kaan et al. (2000) also reported no sustained negativity for an embedded *wh*-contrast similar to our own (see Figure 3 of their paper). There is some precedent in the literature for variation in the distribution of sustained negativities: Notably, Phillips et al. (2005) reported a sustained negativity that was more anteriorly distributed when the dependency spanned a longer distance, and more posteriorly distributed when the dependency spanned a shorter distance. The sizable posterior shift in the distribution of the sustained effect in the current experiment would appear to require a sizable change in the location of the neural generators of the effect. We discuss this variation in further detail in the General Discussion section.

In any case, the change in the distribution of the sustained negativity in the *wh*-dependency control comparison raises some potential questions about how to interpret the lack of a sustained negativity in the adverb condition. Namely, could we conclude from Experiment 2 that the processes that drive the SANs reported in prior literature are not operative in the subordinate cases in general, or is it possible that something about the design or implementation of the experiment blocked the computations that normally elicit SANs? In Experiment 3, we aimed to obtain more data to distinguish between these different possibilities.

### Experiment 3

The goal of Experiment 3 was to re-evaluate the subordinate adverb contrast, given the lack of a SAN for the *wh*-contrast used in Experiment 2. We tested the subordinate comparison again, but this time for the control comparison, we returned to the same matrix *wh*-contrast that elicited a SAN in Experiment 1, and we also used a more similar task (evaluating the fit of a follow-up sentence, rather than the simple comprehension questions used in Experiment 2).

#### Materials

(15)  I think that after Charlotte quit her job, she traveled to Europe.
(16)  I think that today Charlotte quit her job.
(17)  Who did the compliments about the food please?
(18)  Did the compliments about the food please the chef?

Experiment 3 had the same experimental conditions as Experiment 2 but used the matrix *wh*-contrast from Experiment 1 (rather than embedded *wh*-questions). We also made several changes to the adverbial conditions. In Experiment 2, although the temporal adverbials require no syntactic prediction of a second clause, some of those items were in fact continued with a second (optional) clause, which could have acted to induce syntactic prediction on a statistical basis (e.g., if participants developed behavioral strategies throughout the experiment). In Experiment 3 we eliminated those continuations in the temporal adverb condition in order to give ourselves the best chance to observe a positive effect. We also introduced a precursor (e.g., *I think that* …) to the adverbial sentences in order to reduce any noise associated from having a sentence-initial analysis epoch. Because this additional precursor made the sentences longer, we removed one word from the first clause (e.g., *Charlotte quit her job* instead of *Charlotte quit her boring job*).

Thus, as in Experiment 2, the experiment had two main contrasts (4 conditions): a matrix *wh*-contrast (*wh*- vs. *yes*/*no*) and the adverb contrast (subordinating vs. temporal). Unfortunately, due to an error, only 15 trials per condition were included in the adverb contrast, along with the 30 trials per *wh*-condition (each subject saw a total 90 sentences). This error notably reduces our power to detect a subordinate adverbial effect. However, we still believe that the results of this experiment provide useful information for interpreting Experiments 1 and 2, an argument which is supported by the fact that the response observed in the matrix *wh*-contrast (described below) appears strong enough to be detected when considering only 15 trials per condition. The stimuli were again arranged using a Latin square design into two lists and pre-randomized.

#### Procedure

All presentation parameters were the same as Experiments 1 and 2. The behavioral task was similar to that used in Experiment 1. For the adverb conditions, subjects had to decide whether a follow-up was a good continuation of the preceding target sentence (i.e., is it a non-sequitur?). For example, after seeing (15) above, subjects might be shown the follow-up *Traveling to Europe was the most exciting thing she’s done in months*, which would be felicitous (makes sense). Conversely, if they saw *Traveling to Japan was the most exciting thing she’s done in months* as a continuation, then this would be considered a non-sequitur (doesn’t make sense). Subjects were given a probe after every trial. 10 practice items were included at the beginning of the experiment. Subjects were again given feedback on their responses.

#### Participants

A total of 31 participants (15 females, 6 undisclosed, 18–25 years old, mean age 19.8) were recruited for Experiment 3. Participants were compensated with university course credit or pay. All participants were native speakers of English and all were classified as being right-handed by the Edinburgh Handedness Inventory (Oldfield, 1971). Inclusion in analysis required at least 60% of trials to be remaining after artifact rejection and 60% overall behavioral accuracy. 9 participants were excluded based on these parameters, leaving 22 participants (11 females, 5 undisclosed, 18–25 years old, mean age 20.1, all right-handed) for analysis.

#### EEG data acquisition

EEG data acquisition procedures were identical to Experiments 1 and 2.

#### EEG analysis

As in Experiment 1, epochs for the *wh*-contrast were time-locked to the onset of the determiner (*the*) immediately following the auxiliary (*did*) in both conditions and were extracted from −100:2,500 ms (up to but excluding the verb). As in Experiment 2, epochs for the adverb contrast were time-locked to the onset of the adverb in both conditions but were now extracted from −100:2,000 ms in order to avoid comparing ERPs to the final word of the first clause in the subordinate condition to the actual final word of a sentence in the control condition.

Averaged ERPs were formed off-line from clean epochs after pre-processing and artifact rejection. Eye-blink and eye-movement artifacts were identified using the ERPLAB step-function routine with a VEOG threshold of 40 Hz and an HEOG threshold of 40 Hz. Muscular and alpha wave artifacts were identified using the peak-to-peak routine with thresholds ranging between 100 and 130 Hz, based on visual inspection. When necessary, channels were interpolated on a per participant basis. We rejected approximately 14.8% (13 items) of all trials: 16.6% for the *wh*-contrast (15.3% WH, 17.9% YN) and 12.9% for the adverb-contrast (13.7% SUB, 12.1% TEMP). For both contrasts, a 100 ms pre-stimulus baseline and 40 Hz low-pass filter were applied to the waveforms off-line. A list of the specific pre-processing parameters used per participant can be found in the Supplementary Materials.

To evaluate the presence of a sustained effect at anterior electrodes, we followed the same analysis plan outlined for Experiment 1. Again, the ANOVA was conducted on the time-window beginning 750 ms after the word triggering a dependency (the auxiliary or the adverb) and spanned through to the end of the epoch.

#### Experiment 3 results

The overall mean behavioral accuracy was 96% with 4% *SD* (WH: 99%, 1% *SD*; YN: 94%, 4% *SD*; SUB: 94%, 5% *SD*; TEMP: 93%, 6% *SD*).

**Figure 5.**
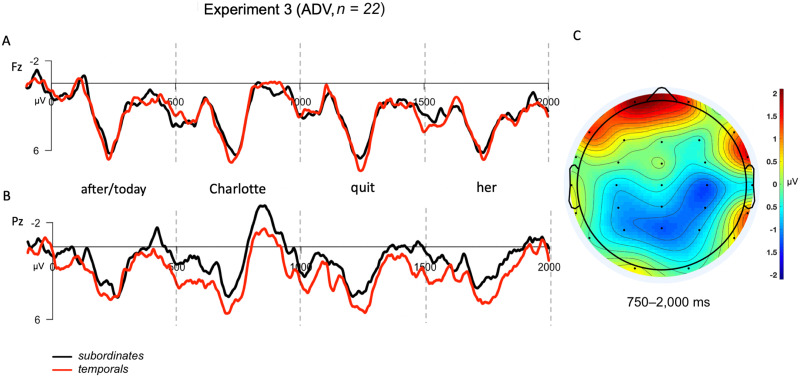
Grand averaged (GA) results for adverb contrast tested in Experiment 3. The epoch is time-locked to the adverb in each condition (*after*/*today*) and spans 2,000 ms into the dependency. (A) GA waveform measured at frontal-central electrode, Fz. (B) GA waveform measured at posterior-central electrode, Pz. (C) Topographic scalp voltage map for region analyzed (750–2,000 ms), showing the voltage difference between the subordinates and temporal adverb conditions (SUB − TEMP). The scale is −2 μV (blue) to +2 μV (red).

For the key adverb contrast, a repeated-measures ANOVA with the parameters outlined above revealed a marginally significant interaction between the two factors (*F*(21) = 3.05, *p* = 0.09) that actually trended in the opposite direction from that predicted (i.e., more negative for the control than for the subordinating condition at anterior electrodes). As shown in Figure 5, while anterior sites appeared more positive for the experimental condition (*M*_SUB,Ant_ = 2.17 μV) than for the control condition (*M*_TEMP,Ant_ = 1.95 μV), the opposite pattern was observed at posterior electrode sites (*M*_SUB,Post_ = 0.91 μV, *M*_TEMP,Post_ = 1.52 μV). In sum, we observed no sign of a SAN for the adverb condition, neither numerically nor statistically. While our failure to detect a SAN could be due to the low number of trials per condition, it is reassuring to note here that the topography of the effect seems similar in distribution to that reported for the adverbial contrast in Experiment 2. We discuss this further in the General Discussion section.

**Figure 6.**
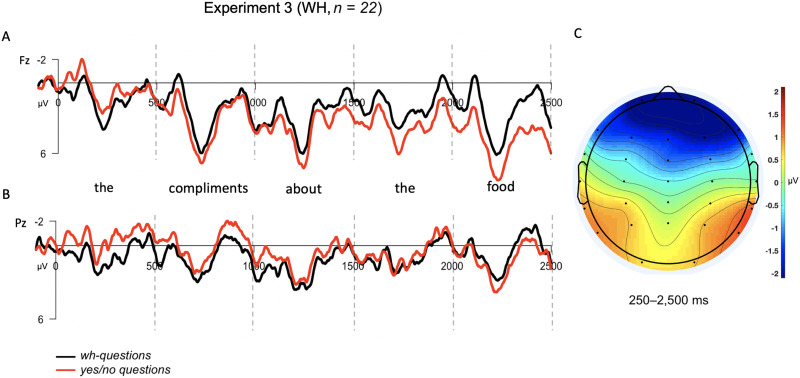
Grand averaged (GA) results for *wh*-contrast used in Experiment 3. The epoch is time-locked to the article (*the*) in both conditions and spans 2,500 ms into the dependency. (A) GA waveform measured at frontal-central electrode, Fz. (B) GA waveform measured at posterior-central electrode, Pz. (C) Topographic scalp voltage map for region analyzed (250–2,500 ms), showing the voltage difference between *wh*- and *yes*/*no* question conditions (WH − YN). The scale is −2 μV (blue) to +2 μV (red).

For the *wh*-contrast, a repeated-measures ANOVA with the parameters outlined in Experiment 1 revealed a significant interaction between the two factors in the expected direction as reported in Experiment 1 (*F*(21) = 24.28, *p* < 0.0001). As shown in Figure 6, anterior sites appeared more negative for the *wh*-condition (*M*_WH,Ant_ = 1.53 μV) than the *yes*/*no* condition (*M*_YN,Ant_ = 2.64 μV), while the opposite was true at posterior sites (*M*_WH,Post_ = 0.79 μV, *M*_YN,Post_ = 0.07 μV). These results closely resemble those reported for Experiment 1, showing a SAN for the *wh*-condition relative to the *yes*/*no* control condition. Moreover, as referenced above, a supplementary analysis considering a random subset of 15 trials in the *wh*-contrast revealed a significant Condition × Anteriority interaction (*F*(21) = 6.88, *p* = 0.016), patterning in the same direction as the main analysis: The *wh*-condition appeared more negative at anterior electrodes (*M*_WH,Ant_ = 1.86 μV, *M*_YN,Ant_ = 3.02 μV), while the *yes*/*no* condition appeared more negative at posterior electrodes (*M*_WH,Post_ = 0.97 μV, *M*_YN,Post_ = 0.31 μV). (See Supplementary Materials for figures and details.)

#### Experiment 3 discussion

In Experiment 3 we tested the matrix *wh*-contrast that elicited a SAN in Experiment 1 and the subordinate contrast from Experiment 2 in the same experimental session, given the lack of a SAN for the *wh*-contrast used as a response localizer in Experiment 2. As expected, the results for the *wh*-contrast in Experiment 3 patterned with those reported for Experiment 1: At anterior electrodes, the long-distance dependency *wh*-condition elicited more negative responses than control conditions throughout the dependency time-window. On the other hand, the results for the adverb contrast again patterned with those of Experiment 2, showing no SAN for the prediction (subordinate) condition relative to the control condition, but only a weak posterior negativity. We interpret these results as evidence against the hypothesis that SANs index the memory maintenance of a syntactic prediction during online sentence processing. Interestingly, the posterior negativity in the adverbial condition drove a marginally significant interaction in Experiment 3, and it is worth noting that the topography of the adverbial contrast somewhat resembles the significant sustained posterior negativity reported for the embedded *wh*-contrast in Experiment 2. We will reserve further discussion of these posterior effects for the General Discussion.

## GENERAL DISCUSSION

In this study, we evaluated whether the SAN—a sustained ERP with anterior distribution and negative polarity that is typically attributed to working memory resources involved in carrying forward a *wh*-filler in memory—can be instead attributed to the carrying forward of syntactic predictions. Because the use of filler–gap dependencies makes it impossible to distinguish these different hypotheses, here we explore the case of subordinate sentences in which a second clause can be predicted in the absence of a filler–gap dependency.

We conducted three experiments aimed at evaluating the structural prediction account (summarized in Table 1). In Experiment 1 (*n* = 14), we showed that simple matrix *wh*-dependencies elicit SANs relative to *yes*/*no* questions with no dependency. In Experiment 2, we failed to observe a SAN for either the embedded *wh*-dependency contrast or the novel subordinate contrast (*n* = 22). Given the surprising lack of a SAN for the *wh*-contrast in Experiment 2 (detailed above), we conducted Experiment 3 to re-evaluate SANs for matrix *wh*-dependencies and the subordinate contrast in a new set of participants (*n* = 22). In Experiment 3, we again observed a SAN for the matrix *wh*-contrast, but there was no measurable effect for the subordinate contrast. Taken together, these results indicate that we failed to find evidence supporting our structural prediction account: Sentences allowing more syntactic prediction failed to reliably elicit a SAN when compared to sentences with less predicted structure. We discuss the implications of these results in detail below.

**Table 1.** Summary of materials, results, and discussion for each of the three experiments in this study.

**Experiment**		**Stimuli**	**Discussion**
**1** (*n* = 14)	**WH** 🗹	What did the commentary from the spokesman interrupt?	The SAN for simple matrix *wh*-questions is in line with prior work.
		Did the commentary from the spokesman interrupt the game?	
**2** (*n* = 22)	**WH** 🛇	John wondered what the commentary from the spokesman interrupted.	The lack of SAN for subordinate contrast suggests SAN does not index syntactic prediction per se. However, the lack of SAN for the embedded *wh*-contrast and the observed posterior effect are surprising given prior literature.
		John wondered whether the commentary from the spokesman interrupted the game.	
	**ADV** 🛇	After Charlotte quit her boring job, she traveled to Europe like she always wanted to.	
		Today Charlotte quit her boring job and traveled to Europe like she always wanted to.	
**3** (*n* = 22)	**WH** 🗹	What did the commentary from the spokesman interrupt?	Results confirm SAN for simple matrix *wh*-questions from Experiment 1. The lack of SAN for the ADV contrast suggests SAN is not an index of syntactic prediction per se.
		Did the commentary from the spokesman interrupt the game?	
	**ADV** 🛇	After Charlotte quit her job, she traveled to Europe like she always wanted to.	
		Today Charlotte quit her job.	

*Note*. WH = *wh*-contrast; ADV = adverb contrast; 🗹 = SAN; 🛇 = no SAN.

### Evidence Against a Syntactic Prediction Account of the SAN

The primary aim of the current study was to test the hypothesis that the SANs reported for *wh*-dependencies in the prior literature might reflect the maintenance of syntactic prediction rather than the working memory storage of features of the *wh*-filler. This hypothesis would have provided an account for Fiebach et al.’s (2002) finding of a SAN contrast between *wh*-object and *wh*-subject constructions in which the distance between the *wh*-filler and the licensing verb were matched, but the amount of syntactic structure predicted was not. However, across two experiments, we found no positive evidence for the syntactic prediction account. We observed no evidence for a SAN in a new manipulation of predicted syntactic structure that focused on the period following a subordinate adverbial that generates a prediction for a subsequent matrix clause. Although we had reduced power to detect the contrast in Experiment 3 because of an experimenter error that resulted in fewer items per condition, we saw no numerical or statistical evidence of a SAN in either experiment; instead, both experiments showed a weak tendency towards a posterior negativity.

One notable, potential caveat about this conclusion is that our design required assumptions about what syntactic material is predicted at a given point in the sentence, and much research in sentence processing has shown that this is challenging to establish (see Gibson, 1998, for a classic discussion). It is similarly challenging to establish the cost function; how much syntactic material equals one unit of cost? For example, unaddressed syntactic predictions abound in our materials, such as the predictive dependency between the tense on the auxiliary (*did*) and the bare form of the main verb (*leave*) in the *yes*/*no* questions. Does this morphosyntactic prediction exert the same memory burden as a prediction for a clause, or a prediction for a transitive verb phrase? Current consensus knowledge about the parsing system does not provide the answer, and therefore it would be possible to maintain the syntactic prediction account of the SAN in the face of these data by arguing that our assumptions were incorrect about the syntactic predictions and their cost in the adverbial sentence conditions. However, we believe that the absence of a SAN in the embedded *wh*-question cases, discussed next, deals an even more severe blow to syntactic accounts of the SAN.

### Evidence Against Traditional Syntactic Working Memory Accounts of the SAN

While we were able to replicate the SAN response in some *wh*-dependency configurations, to our surprise the overall pattern of results in these conditions is also challenging to explain for the standard syntactic working memory accounts of the SAN. Experiments 1 and 3 provide evidence for the existence of a SAN for matrix *wh*-questions similar to those originally used in Kluender and Kutas (1993) and recently used in Sprouse et al. (2021). Given that SAN effects for *wh*-dependencies have received surprisingly little attention in the language ERP literature in the last 20 years, and have particular relevance for influential recent sentence processing models that reject the idea of sustained cost working memory (e.g., Lewis & Vasishth, 2005; McElree et al., 2003; see Sprouse et al., 2021, for a detailed discussion), it is reassuring to confirm that the SAN can be reliably elicited by multiple research groups.

The results of Experiments 1 and 3, together with converging results from Sprouse et al. (2021), indicate that matrix *wh*-question materials can be effectively used as a localizer against which SANs elicited by other constructions can be confirmed. However, in Experiment 2 we failed to observe a SAN response for the same *wh*-question materials when we made the theoretically-minor alteration of turning them into embedded *wh*-questions. This would not be predicted by standard syntactic working memory accounts of the SAN because there is no obvious sense in which embedding the dependency under a complementizer changes the long-distance syntactic dependency between the *wh*-filler and the gap position. One possible explanation is that the absence of a SAN in the embedded *wh*-condition in Experiment 2 was simply a false negative, perhaps driven by properties of the comprehension task. However, in a subsequent recent experiment, Sprouse et al. (2021) replicated the absence of a SAN for these embedded *wh*- items using an acceptability judgment task in a within-subjects design. Another possibility is that our control condition *whether* actually instantiates an invisible filler–gap dependency. However, in their follow-up experiment, Sprouse et al. (2021) failed to observe a SAN in embedded *wh*-items when using *if* as the control complementizer. Therefore, there appears to be reasonably good evidence for some sort of contrast between the matrix and embedded *wh*-contexts in generating a SAN response. However, because several of the most well-known prior studies demonstrating SAN effects for *wh*-dependencies used embedded questions (Fiebach et al., 2002; Phillips et al., 2005; although cf. Kaan et al., 2000, who failed to observe a phasic anterior negativity to an embedded *wh*-contrast similar to our own) and RCs (King & Kutas, 1995), the computations underlying the SAN are unlikely to be exclusive to matrix *wh*-questions.

### Towards a Non-Syntactic, Discourse-Based Account of the SAN?

Although much more work will be needed to evaluate alternative explanations for these emerging generalizations about which sentence configurations elicit SANs and which do not, our own assessment is that we should seriously consider the possibility that the computations generating the SAN are interpretive operations, not syntactic ones. For example, one potentially relevant difference between matrix and embedded *wh*-questions is sentential force: Matrix *wh*-questions are questions that request information that is presumed to be available (i.e., they presuppose the existence of the entities or events that are being asked about), while embedded *wh*-questions are indirect questions (and therefore effectively statements) that provide new information. Therefore, the SAN could be tracking some computations that align with the difference between the two types of “questions.”

Another possibility is that differences in non-syntactic properties of sentence materials, such as number or type of discourse referents, are responsible for the variation in the SAN across studies with embedded *wh*-dependencies. For example, we note that the other embedded *wh*-dependency study we are aware of that did not observe a SAN (Kaan et al., 2000) used simple sentences with proper name matrix subjects, just as we did (e.g., *Emily wondered whether the performer in the concert* … (p. 164)). On the other hand, Phillips et al. (2005) used definite NPs (e.g., *The lieutenant knew which accomplice the detective hoped* … (p. 410, Table 1)). While this difference may seem inconsequential, other work has suggested that sentences with multiple definite NPs induce greater processing difficulty than those with a mix of proper names or pronouns (Gordon et al., 2004), perhaps because the features of those referents are more easily confusable in working memory.

Recent work emphasizes discourse and pragmatic factors in modulating the presence/absence of the SAN: specifically, Yano and Koizumi (2018, 2021) propose an account that interprets the SAN as reflecting the active manipulation of a discourse representation to accommodate new information. In their studies, they manipulated the “givenness” of an argument (a referent NP) in sentences with non-canonical word order to evaluate the role effect of pragmatic factors on the SAN. Consistent with their “discourse representation” account, they showed that the SAN for object-first (filler–gap) sentences—which presuppose the availability of a shared referent/argument—can be alleviated by first introducing referents/arguments via a discourse context, rendering the use of these kinds of non-canonical constructions felicitous. Thus, it is possible that matrix *wh*-questions introduced without any supportive context elicit a SAN because they require comprehenders to accommodate presupposed information into their discourse representation in order to evaluate the question, while the lack of a SAN for embedded *wh*-sentences reflects the fact that they provide (rather than elicit) information. Yano and Koizumi (2021) suggest that SAN effects for object RCs (King & Kutas, 1995) might also reflect presupposition failures; the subject of an object RC should be “given” by prior discourse in order to act felicitously as a restrictor of the head noun referent, and when it is not, difficulty ensues. Another version of this idea is that an embedding clause generates a richer discourse structure representation to which incoming referents can be bound, resulting in a more efficient working memory discourse representation for encoding the *wh*-referent than in the bare matrix *wh*-question where there is nothing to relate it to.

### Sustained Neural Responses as a Mechanism for Working Memory in Language

Finally, we can consider the broader implications of these results for the idea that working memory in language depends on sustained neural activity. As the absence of a SAN in the embedded *wh*-condition appears inconsistent with the syntactic working memory account of the SAN, perhaps the reader might be tempted to conclude that these findings support “activity silent” theories of working memory, in which encoding of information for ongoing computations does not require active maintenance through ongoing neural activity but is instead supported by standard long-term memory encoding operations (e.g., Lewis & Vasishth, 2005; Jonides et al., 2008). However, we think this conclusion would be too strong. Our study was aimed at determining whether a particular sustained response—a sustained negativity over anterior electrodes—reflected maintenance of syntactic material. A different design and analysis approach would be needed to conduct a more exhaustive search for sustained working memory responses.

In fact, in the current data set we did observe a sustained response with a different profile for embedded *wh*-sentences: a sustained negativity with a posterior distribution. One possible explanation for the posterior negativity that unifies it with the SANs we observed for matrix *wh*-dependencies is that sustained neural activity is a general mechanism through which information can be carried forward in working memory, one which can be used by different brain regions depending on the specifics of the information being maintained or task demands (e.g., one region that maintains syntactic information, a different region that maintains discourse representations). While much more work would have to be done to understand this variation in topography, and what exactly different brain regions are encoding via sustained activity, such an interpretation would be consistent with some behavioral work arguing for active maintenance mechanisms in syntactic dependencies (Goodall, 2015; Wagers & Phillips, 2014) and with the broader hypothesis that attributes sustained neural activity to working memory (e.g., Constantinidis et al., 2018). This interpretation could also be compatible with fMRI work in which both linguistic structure size and non-linguistic meaning are manipulated, and where the results suggest distinct effects of connectedness in linguistic and non-linguistic conceptual regions (e.g., Matchin et al., 2017; Pallier et al., 2011). By the same token, it is intriguing that the subordinate adverbial conditions showed non-significant or marginally significant trends towards a posterior negativity in both Experiment 2 and Experiment 3. We also note that Phillips et al. (2005) observed a more posterior distribution for the sustained negativity in their short distance dependency relative to their long-distance dependency, although in our study dependency length was constant and as such cannot explain the contrast in distribution between matrix and embedded effects.

These considerations suggest the intriguing hypothesis of a common neural mechanism—sustained neural activity for working memory—implemented separately for different information types: sustained activity in the non-linguistic referential system manifesting as anterior negativities in EEG, sustained activity in the linguistic syntactic parsing system manifesting as posterior negativities in EEG. We caution that since we had no a priori hypotheses about posterior sustained responses going into this study, we cannot draw strong conclusions about their role in working memory maintenance here. However, we believe that establishing the role of sustained neural activity in working memory representations for language comprehension should be a central priority of neuroscience of language research in years to come.

Our results suggest that the SAN is not straightforwardly attributable to the working memory resources required to maintain available syntactic predictions, nor to computations required for processing all *wh*-dependencies. Instead, it is possible that the SAN is reflective of non-linguistic discourse or referential processes engaged by sentence comprehension. Given the broader theoretical implications, these results provide the foundations for future work clarifying the function of sustained neural responses, and the SAN more specifically, across a variety of sentence constructions (e.g., with and without *wh*-dependencies), task demands (e.g., with and without context), and neural measures (e.g., event-related vs. oscillatory). Finally, we believe this work should be considered part of a growing research enterprise that seeks to integrate findings from across the working memory and discourse processing literatures.

## ACKNOWLEDGMENTS

We thank Lalitha Balachandran, Maggie Kandel, Macie McKitrick, Stephanie Pomrenke, Katte Luckhurst, Fenton Ingram, Anna Liddle, and Anna Namyst for help with data collection. Thanks to Jon Sprouse for comments on an earlier version of this manuscript. We are also very grateful to anonymous reviewers and an editor for their insightful comments and suggestions. This material is based upon work supported by the National Science Foundation under Grant No. BCS-1749407.

## FUNDING INFORMATION

Ellen Lau, National Science Foundation (https://dx.doi.org/10.13039/100000001), Award ID: BCS-1749407.

## AUTHOR CONTRIBUTIONS

**Aura A. L. Cruz Heredia**: Conceptualization: Lead; Data curation: Lead; Formal analysis: Lead; Investigation: Lead; Methodology: Lead; Project administration: Lead; Writing – original draft: Lead; Writing – review & editing: Lead. **Bethany Dickerson**: Conceptualization: Supporting; Writing – review & editing: Supporting. **Ellen Lau**: Conceptualization: Equal; Funding acquisition: Lead; Supervision: Equal; Writing – original draft: Equal; Writing – review & editing: Equal.

## TECHNICAL TERMS

Working memory: Can be thought of as the short-term retention of task-relevant information/stimuli.
Long-distance dependencies: Structural or referential “linkages” between two non-adjacent elements in a sentence (e.g., filler and gap, name and pronoun).
Sustained neural activity: Persistent activity that spans a working memory task/dependency region.
Syntactic prediction: The hypothesis that individuals actively construct (top-down) expectations for structured elements (e.g., phrases).
