# Supplementary material for: Towards Understanding Sustained Neural Activity Across Syntactic Dependencies
Source: Neurobiol Lang (Camb). 2022 Feb 10;3(1):87–108. doi: 10.1162/nol_a_00050 (PMC10158612; doi:10.1162/nol_a_00050)
Supplement: Supplementary file 1 [file nol-3-1-87-s001.pdf]

Supplementary Materials

Author's Note: For higher resolution versions of these figures, please email

aurach@sas.upenn.edu

Experiment 1: Wh-Question Contrast (n=14)

A. Pre-Processing Information & Behavioral Task Accuracy (Exp 1, WH)

Peak-to-Peak Threshold (μV)	HEOG Threshold (μV)	VEOG Threshold (μV)	Electrodes Interpolated	Behavioral Accuracy	Number of Trials Kept after Artifact Rejection (WH)	Number of Trials Kept after Artifact Rejection (YN)	Overall Percentage of Trials Kept after Artifact Rejection
100	40	25		90.0%	26	25	85.0%
100	40	25		90.0%	28	29	95.0%
100	40	25		81.7%	30	30	100.0%
100	40	25		93.3%	30	30	100.0%
100	40	25	O1	83.3%	24	28	86.7%
100	40	25	O1, O2	75.0%	25	26	85.0%
100	40	25	O2	95.0%	28	30	96.7%
100	40	25		91.7%	29	30	98.3%
100	40	25		78.3%	29	27	93.3%
100	40	25		96.7%	29	29	96.7%
100	40	25		96.7%	27	29	93.3%
100	40	25	FT7	76.7%	24	28	86.7%
100	40	25	FP1	88.3%	30	27	95.0%
100	40	25	FT7	85.0%	24	24	80.0%

B. Word-by-Word Scalp Voltage Maps (Exp 1, WH)

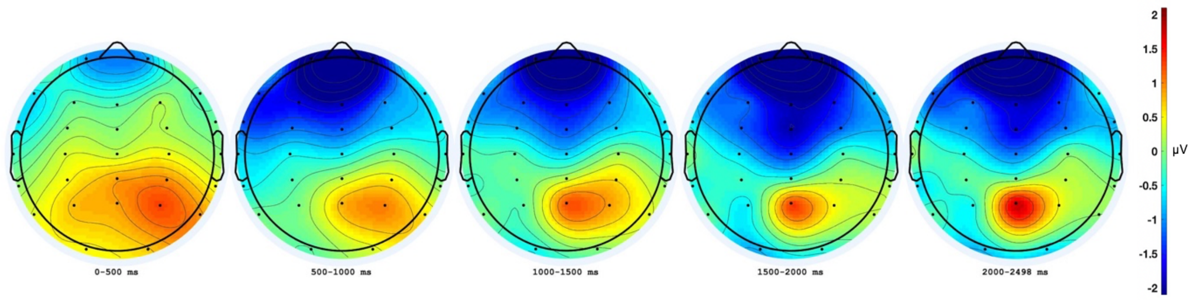

C. Grand-Averaged Waveforms for 29 electrodes (Exp 1, WH)

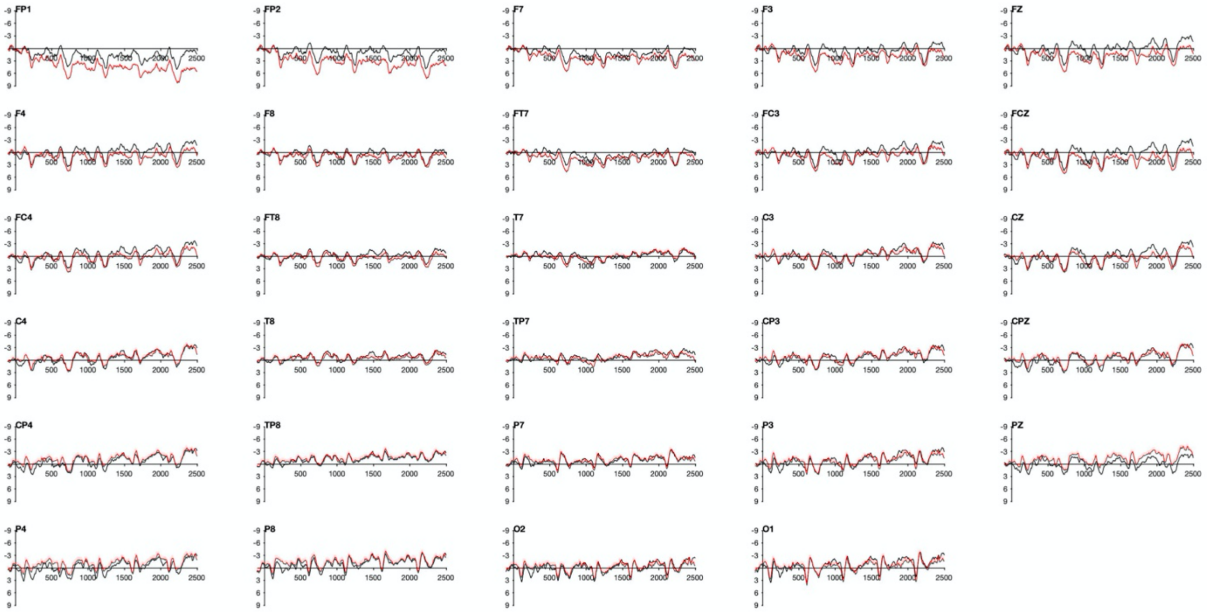

<u>Experiment 2: Adverb Contrast (n=22)</u>

D. Pre-Processing Information & Behavioral Task Accuracy (Exp 2, ADV)

Peak-to-Peak Threshold (µV)	HEOG Threshold (µV)	VEOG Threshold (µV)	Electrodes Interpolated	Behavioral Accuracy	Number of Trials Kept after Artifact Rejection (SUB)	Number of Trials Kept after Artifact Rejection (TEMP)	Overall Percentage of Trials Kept after Artifact Rejection
100	40	25		95.0%	29	29	96.7%
100	40	25		92.5%	23	19	70.0%
100	40	25		100.0%	23	25	80.0%
100	45	35		95.0%	21	22	71.7%
100	40	25		98.6%	25	25	83.3%
130	40	25	O1	92.5%	26	23	81.7%
125	40	25		95.0%	23	27	83.3%
100	40	25		98.6%	23	20	71.7%
100	40	25	T8,FT8,TP7,F8	95.0%	28	26	90.0%
100	40	25		100.0%	26	26	86.7%
130	40	25	O2	92.5%	29	29	96.7%
100	40	25		95.0%	25	24	81.7%
110	40	25	F8, T8	92.5%	20	22	70.0%
100	40	25		93.6%	21	23	73.3%
100	40	25		93.6%	23	27	83.3%
100	40	25		100.0%	24	23	78.3%
120	40	25	T8	95.8%	22	21	71.7%
115	40	25		98.6%	25	25	83.3%
100	40	25		95.0%	22	22	73.3%
100	40	25	FP2	92.5%	24	24	80.0%
100	40	25		93.6%	29	27	93.3%
135	40	35	FP2	95.0%	21	21	70.0%

E. Word-by-Word Scalp Voltage Maps (Exp 2, ADV)

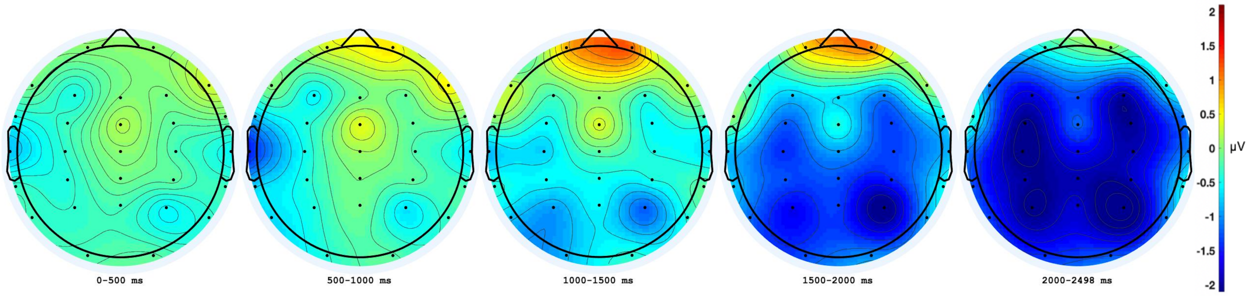

F. Grand-Averaged Waveforms for 29 electrodes (Exp 2, ADV)

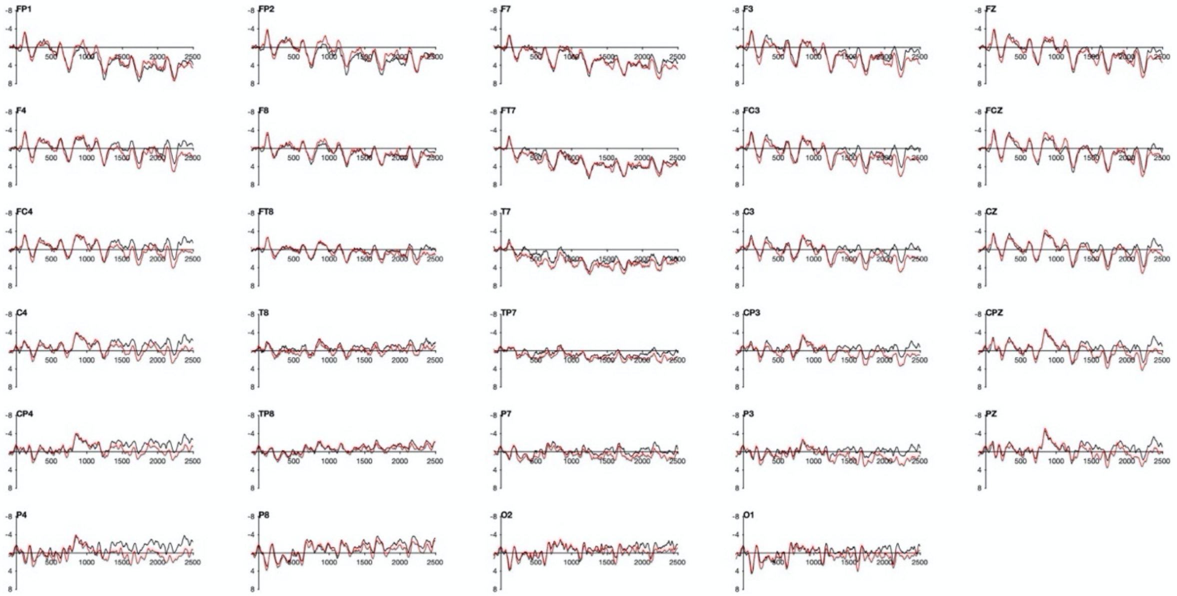

Experiment 2: Wh-Question Contrast (n=22)

G. Pre-Processing Information & Behavioral Task Accuracy (Exp 2, WH)

Peak-to-Peak Threshold (μV)	HEOG Threshold (μV)	VEOG Threshold (μV)	Electrodes Interpolated	Behavioral Accuracy	Number of Trials Kept after Artifact Rejection (WH)	Number of Trials Kept after Artifact Rejection (YN)	Overall Percentage of Trials Kept after Artifact Rejection
100	40	25		100.0%	27	30	95.0%
115	40	25		100.0%	21	23	73.3%
100	40	25		100.0%	25	27	86.7%
100	40	25		100.0%	27	27	90.0%
100	40	25		97.2%	26	24	83.3%
100	40	25	O1	100.0%	20	23	71.7%
100	40	25	T8, FT8	100.0%	23	20	71.7%
100	40	25		97.2%	26	23	81.7%
100	40	25		100.0%	29	29	96.7%
100	40	25		100.0%	28	29	95.0%
100	40	25		100.0%	30	30	100.0%
100	40	25		100.0%	27	25	86.7%
100	40	25	O2, T8	100.0%	23	25	80.0%
100	40	25		97.2%	23	24	78.3%
100	40	25		97.2%	26	26	86.7%
100	40	25		100.0%	26	28	90.0%
100	40	25	T8	91.7%	24	24	80.0%
100	40	25		97.2%	29	29	96.7%
100	40	25		100.0%	27	28	91.7%
100	40	25	FP2	100.0%	25	23	80.0%
100	40	25		97.2%	29	28	95.0%
135	40	30	FP2, F8	100.0%	19	23	70.0%

H. Word-by-Word Scalp Voltage Maps (Exp 2, WH

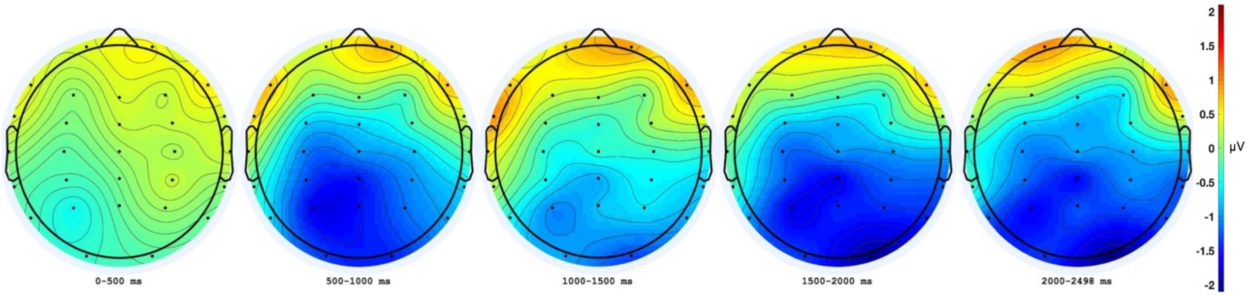

I. Grand-Averaged Waveforms for 29 electrodes (Exp 2, WH)

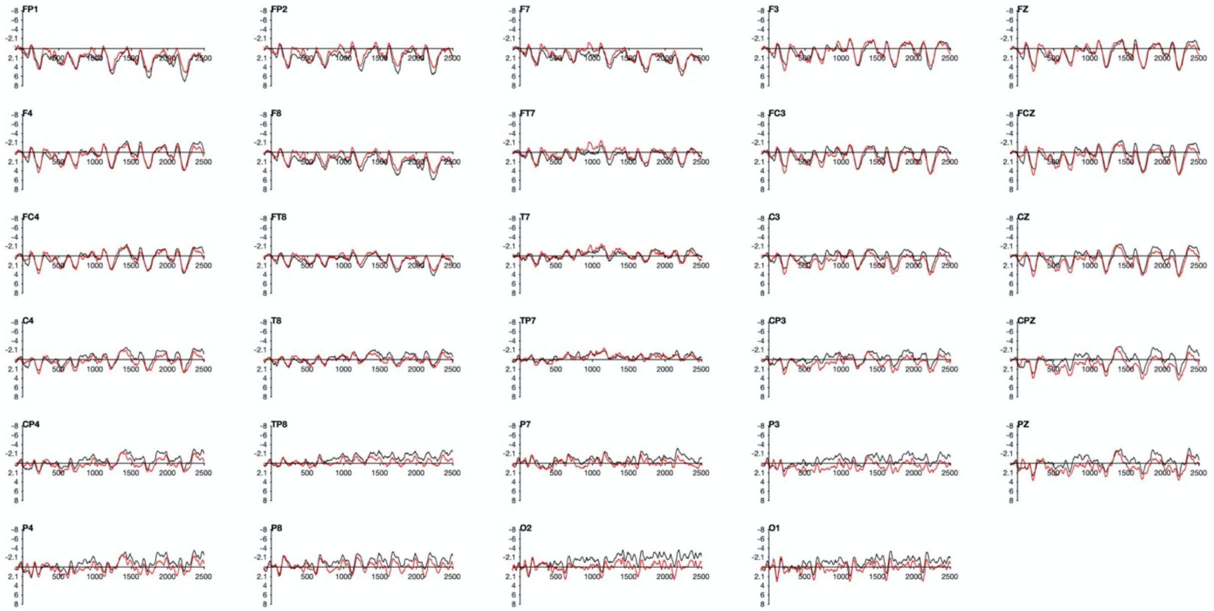

Experiment 3: Adverb Contrast (n=22)

J. Pre-Processing Information & Behavioral Task Accuracy (Exp 3, ADV)

Peak-to-Peak Threshold (μV)	HEOG Threshold (μV)	VEOG Threshold (μV)	Electrodes Interpolated	Behavioral Accuracy	Number of Trials Kept after Artifact Rejection (SUB)	Number of Trials Kept after Artifact Rejection (TEMP)	Overall Percentage of Trials Kept after Artifact Rejection
100	40	25		88.0%	12	12	80.0%
100	40	25		100.0%	13	14	90.0%
100	40	25		75.0%	13	15	93.0%
100	40	25		88.0%	15	15	100.0%
100	40	25		63.0%	12	12	80.0%
100	40	25		88.0%	13	14	90.0%
100	40	25		75.0%	14	12	87.0%
100	40	25		88.0%	14	13	90.0%
100	40	25	T8, FT8, TP7, F8	100.0%	13	14	90.0%
100	40	25		100.0%	13	14	90.0%
130	40	25	O2	88.0%	11	11	73.3%
100	40	25		63.0%	14	15	97.0%
100	40	25	O1, T8, F8	75.0%	11	12	77.0%
100	40	25		88.0%	12	12	80.0%
100	40	25		88.0%	15	14	97.0%
100	40	25		100.0%	13	12	83.0%
100	40	25		88.0%	13	13	87.0%
115	40	25		88.0%	11	10	70.0%
100	40	25		88.0%	14	15	97.0%
100	40	25		88.0%	13	15	93.0%
100	40	25		88.0%	14	13	90.0%
100	40	25	O1, O2	87.3%	13	14	90.0%

K. Word-by-Word Scalp Voltage Maps (Exp 3, ADV)

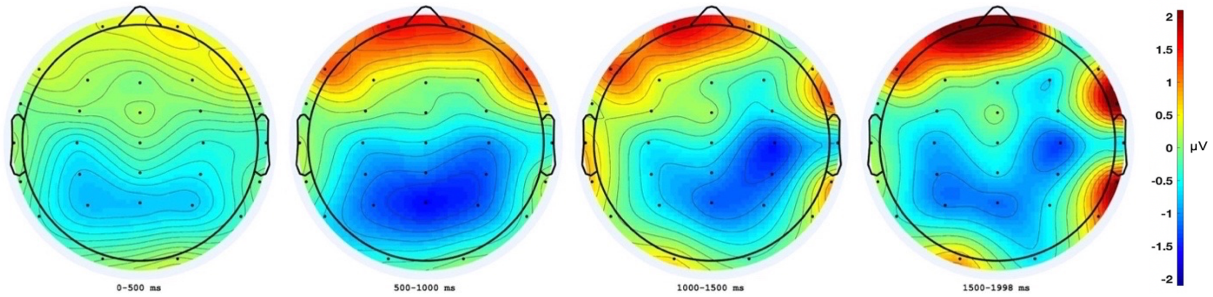

L. Grand-Averaged Waveforms for 29 electrodes (Exp 3, ADV)

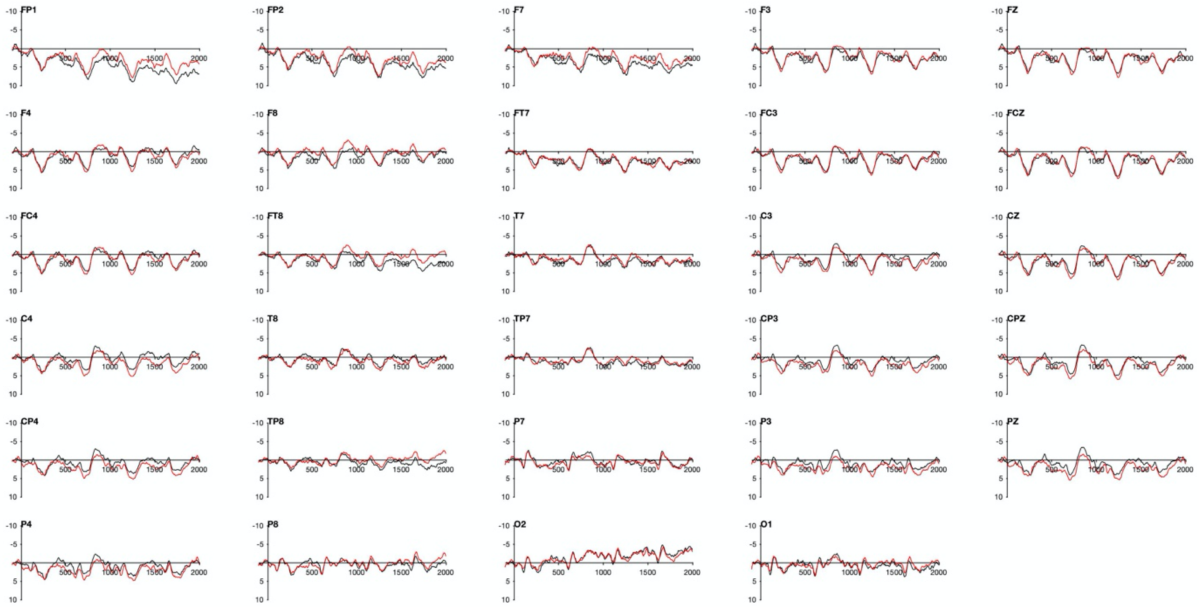

<u>Experiment 3: Wh-Question Contrast (n=22)</u>

M. Pre-Processing Information & Behavioral Task Accuracy (Exp 3, WH)

Peak-to-Peak Threshold (μV)	HEOG Threshold (μV)	VEOG Threshold (μV)	Electrodes Interpolated	Behavioral Accuracy	Number of Trials Kept after Artifact Rejection (WH)	Number of Trials Kept after Artifact Rejection (YN)	Overall Percentage of Trials Kept after Artifact Rejection
100	40	25	O2	82.1%	24	25	82.0%
100	40	25	O2	87.5%	28	24	87.0%
100	40	25	O2	84.4%	29	29	97.0%
100	40	25	O2	80.2%	29	30	98.0%
125	40	35		78.1%	24	21	75.0%
100	40	25	O2	84.4%	28	27	92.0%
100	40	25		86.5%	27	24	85.0%
100	40	25		75.0%	25	21	77.0%
100	40	25	T8, FT8, TP7, F8	84.4%	25	28	88.0%
100	40	25		83.3%	26	26	87.0%
100	40	25	O2	69.8%	23	19	70.0%
100	40	25		77.1%	26	29	92.0%
100	40	25	O1, T8, F8	83.3%	22	23	75.0%
125	40	35		81.3%	22	21	71.7%
100	40	25		85.4%	27	26	88.0%
100	40	25		84.4%	24	22	77.0%
100	40	25		82.9%	24	22	77.0%
100	40	25		75.5%	24	22	77.0%
100	40	25		68.8%	25	30	92.0%
100	40	25		83.3%	28	26	90.0%
100	40	25		74.7%	26	26	87.0%
100	40	25	O1, O2	86.7%	29	28	95.0%

N. Word-by-Word Scalp Voltage Maps (Exp 3, WH)

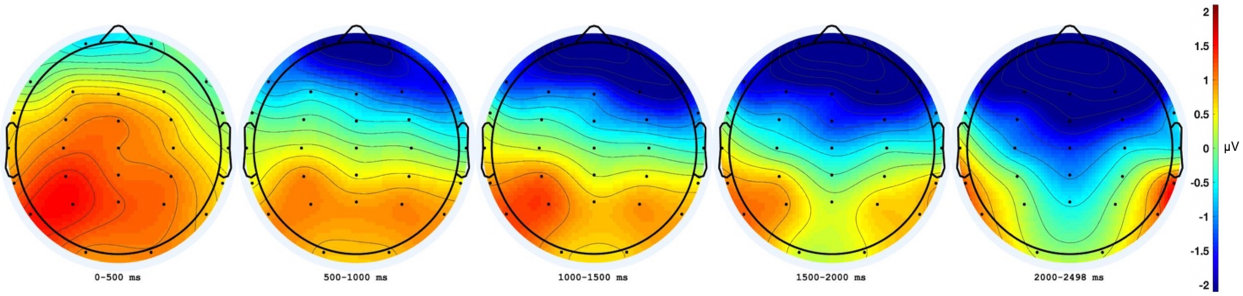

O. Grand-Averaged Waveforms for 29 electrodes (Exp 3, WH)

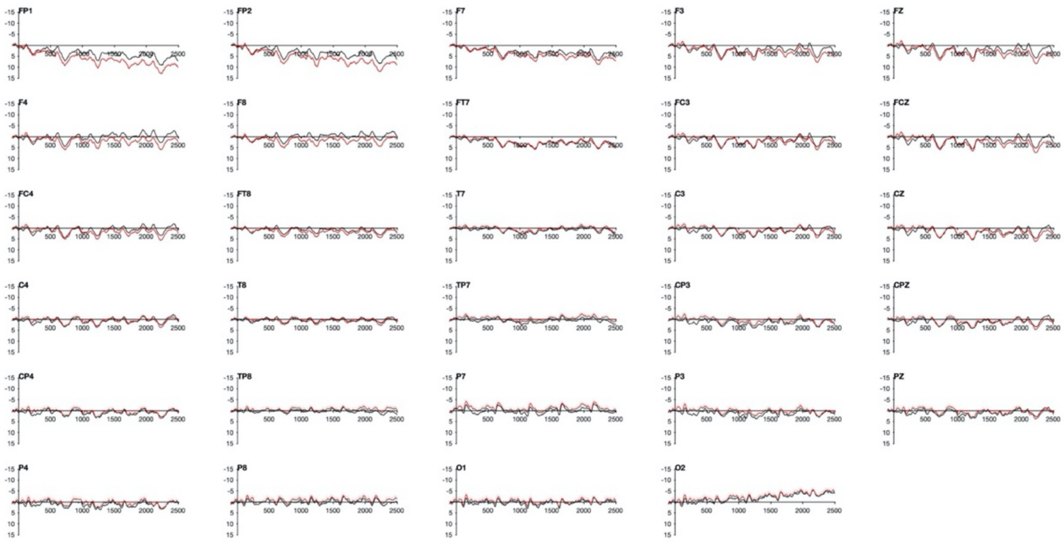

Experiment 3: Wh-Questions Supplementary Analysis (SA) (n=22)

Supplementary analysis considering a random subset of 15 trials in the wh-contrast.

P. Grand averaged (GA) results for the matrix wh-contrast used in Experiment 3-SA.

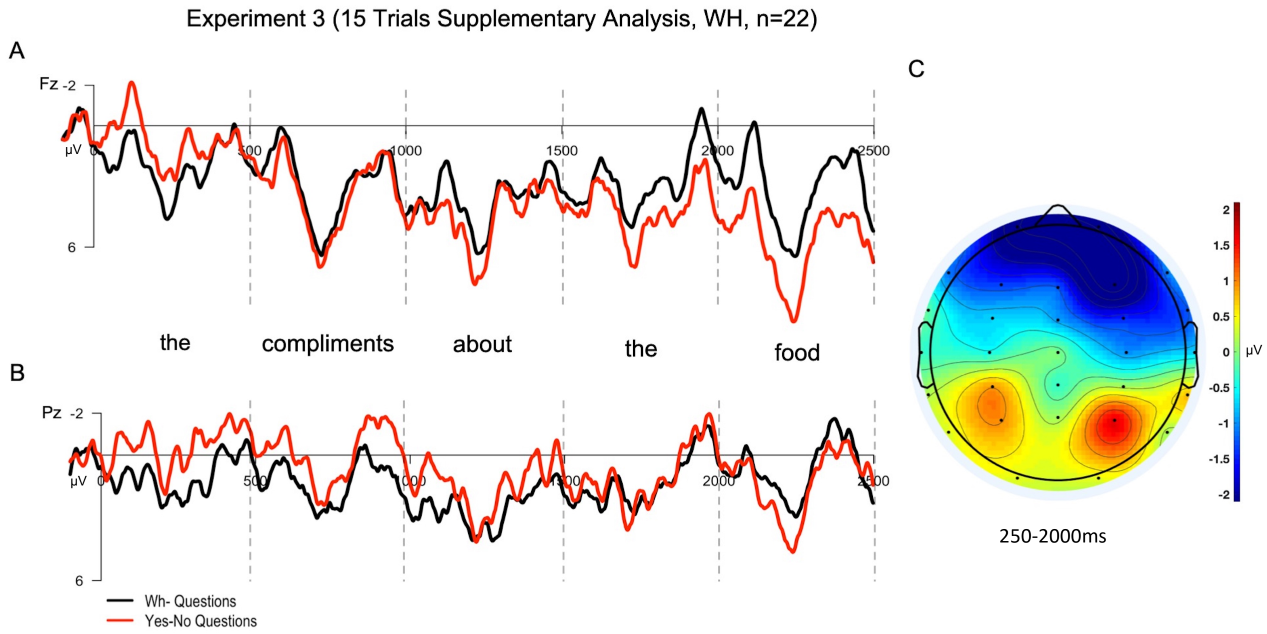

The epoch is time-locked to the determiner ('the') and spans 2500ms into the dependency (up to but excluding the verb). (A) GA waveform measured at frontal-central electrode, Fz. (B) GA waveform measured at posterior-central electrode, Pz. (C) Topographic scalp voltage map for region analyzed (250ms-2500ms), showing the voltage difference between wh- and yes-no question conditions (WH – YN). The scale is -2μV (blue) to +2μV (red).

Q. Pre-Processing Information & Behavioral Task Accuracy (Exp 3-SA, WH)

Peak-to-Peak Threshold (μV)	HEOG Threshold (μV)	VEOG Threshold (μV)	Electrodes Interpolated	Behavioral Accuracy	Number of Trials Kept after Artifact Rejection (WH)	Number of Trials Kept after Artifact Rejection (YN)	Overall Percentage of Trials Kept after Artifact Rejection
100	40	25	O2	82.1%	15	14	96.7%
100	40	25	O2	87.5%	15	12	90.0%
100	40	25	O2	84.4%	15	14	96.7%
100	40	25	O2	80.2%	15	15	100.0%
125	40	35		78.1%	12	13	83.3%
100	40	25	O2	84.4%	14	12	86.7%
100	40	25		86.5%	15	14	96.7%
100	40	25		75.0%	14	9	76.7%
100	40	25	T8, FT8, TP7, F8	84.4%	13	14	90.0%
100	40	25		83.3%	14	13	90.0%
100	40	40	O2	69.8%	12	10	73.3%
100	40	25		77.1%	14	15	96.7%
100	40	25	O1, T8, F8	83.3%	12	11	76.7%
125	40	35		81.3%	12	9	70.0%
100	40	25		85.4%	13	12	83.3%
100	40	25		84.4%	14	12	86.7%
100	40	25		82.9%	12	11	76.7%
100	40	25		75.5%	12	11	76.7%
100	40	25		68.8%	15	15	100.0%
100	40	25		83.3%	14	13	90.0%
100	40	25		74.7%	12	12	80.0%
100	40	25	O1, O2	87.3%	14	14	93.3%

R. Word-by-Word Scalp Voltage Maps (Exp 3-SA, WH)

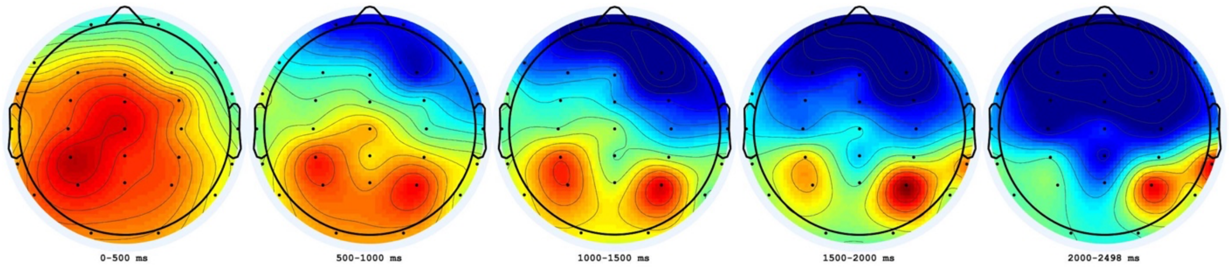

S. Grand-Averaged Waveforms for 29 electrodes (Exp 3-SA, WH)

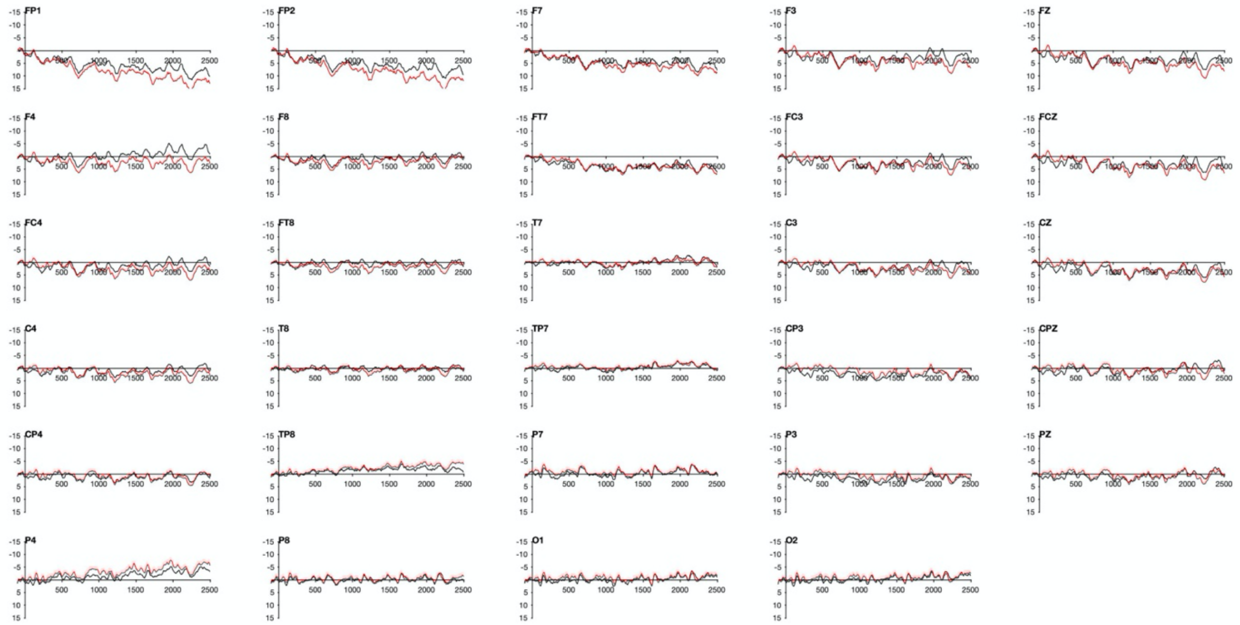

T. Results (Exp 3-SA, WH contrast)

Condition	
1 (WH)	1.41753 μV
2 (YN)	1.66505 μV
Main Effect: F(21) = 1.498678, p = 0.736391842	

Anteriority	
Anterior	2.43974 μV
Posterior	0.64285μV
Main Effect: F(21) = 13.6810133, p = 0.001331941*	

	Anterior	Posterior
Wh- Questions	1.86294 μV	0.97212 μV
Yes-No Questions	3.01653 μV	0.31358 μV
**Condition x Anteriority Interaction Effect: F(21) = 6.8768641, p = 0.015915456***		
